# Dominance of the E198A Mutation and Emergence of Co-Selection in Benzimidazole-Resistant *Haemonchus contortus* from Northwestern China

**DOI:** 10.3390/vetsci13060603

**Published:** 2026-06-21

**Authors:** Waresi Tuersong, Lianxi Xin, Abudusaimaiti Tuoheti, Ailixire Maimaiti, Dilare Xuekelaiti, Reyilanmu Tuerhong, Wei Zhang, Bayinchahan Gailike, Qingyong Guo, Saifuding Abula

**Affiliations:** 1 College of Veterinary Medicine, Xinjiang Agricultural University, Urumqi 830052, China; 18199306877@163.com (W.T.); 17609922023@163.com (L.X.);; 2 Xinjiang Key Laboratory of New Drug Research and Development for Herbivorous Animals, Urumqi 830052, China; 3 Veterinary Medicine Postdoctoral Research Station of Xinjiang Agricultural University, Urumqi 830052, China; 4 Veterinary Research Institute, Xinjiang Academy of Animal Husbandry, Urumqi 830013, China; 5 Changji University, Changji 831100, China

**Keywords:** *Haemonchus contortus*, benzimidazole resistance, E198A, F200Y, Isotype-1 β-tubulin, Xinjiang, anthelmintic resistance surveillance, pastoral sheep, abattoir-based monitoring

## Abstract

**Background:** Benzimidazole (BZ) resistance in the gastrointestinal nematode *Haemonchus contortus* is a major constraint to sheep production worldwide. However, data on the prevalence and molecular mechanisms of resistance in Yili Prefecture, Xinjiang—a key livestock region in Northwestern China—remain limited. This study aimed to determine the frequency of BZ resistance-associated single nucleotide polymorphisms (SNPs) in *H. contortus* populations from Zhaosu and Tekesi counties. **Methods:** Adult male worms (n = 150) were collected from naturally infected sheep at local abattoirs. Species identity was confirmed morphologically by sequencing the internal transcribed spacer 2 (ITS-2) region. A 385 bp fragment of the isotype-1 β-tubulin gene was amplified and sequenced to detect SNPs at codons 167 (F167Y), 198 (E198A), and 200 (F200Y). **Results:** The F167Y mutation was absent in all individuals. In contrast, the E198A mutation occurred at exceptionally high frequencies, with resistant allele frequencies (RAF) of 64.7% in Zhaosu and 52.7% in Tekesi. The F200Y mutation showed clear geographical variation: it remained low in Zhaosu (RAF = 9.3%) but was substantially higher in Tekesi (RAF = 33.3%). Haplotype analysis revealed that resistance in Zhaosu was driven primarily by the E198A mutation, whereas the Tekesi population exhibited complex patterns of co-selection of both E198A and F200Y, with a high proportion of double-heterozygous individuals (29.3%). **Conclusions:** This study provides comprehensive molecular evidence of severe BZ resistance in *H. contortus* populations from Zhaosu and Tekesi counties, Yili Prefecture. The marked predominance of the E198A mutation, together with the emergence of multi-locus resistance in Tekesi, indicates a rapid escalation of resistance beyond historical levels. These findings suggest that benzimidazoles are likely ineffective in this region and highlight the urgent need to revise local parasite control strategies.

## Simple Summary

Benzimidazole (BZ) resistance in *Haemonchus contortus* severely threatens sheep production worldwide. This study investigated BZ resistance-associated mutations in worm populations from two major pastoral counties in Yili, Xinjiang. We analyzed 150 adult male worms and screened three key single nucleotide polymorphisms (F167Y, E198A, F200Y) of the isotype-1 β-tubulin gene. The F167Y mutation was absent in all samples. The E198A mutation dominated in both populations with high resistant allele frequencies (64.7% and 52.7%). The F200Y mutation showed obvious geographic differences and reached 33.3% in Tekesi, where co-selection of E198A and F200Y was prevalent. Our findings confirm severe BZ resistance in local *H. contortus* populations. Benzimidazoles are no longer effective here, and updated parasite control strategies are urgently required.

## 1. Introduction

Gastrointestinal nematodes (GINs) represent a significant threat to small ruminant production worldwide, causing reduced weight gain, anemia, and mortality. Among these, *Haemonchus contortus* is arguably the most pathogenic and economically damaging species [1]. For decades, the control of haemonchosis has relied heavily on the use of broad-spectrum anthelmintics, particularly benzimidazoles (BZs). However, the intensive and often indiscriminate use of these drugs has led to the widespread emergence of BZ resistance, which is now a global phenomenon threatening the sustainability of sheep farming [2,3].

The mechanism of BZ resistance in trichostrongylid nematodes is well elucidated. It is primarily linked to single nucleotide polymorphisms (SNPs) in the isotype-1 β-tubulin gene, which prevent the binding of the drug to its target [4]. Three specific SNPs are principally associated with resistance: F167Y (TTC to TAC at codon 167), E198A (GAA to GCA at codon 198), and F200Y (TTC to TAC at codon 200) [5,6]. While the F200Y mutation is the most widely distributed resistance allele globally, emerging evidence suggests that the E198A mutation may play a more prominent role in specific regions, particularly in China [7,8,9]. Understanding the local prevalence of these alleles is crucial for designing effective control strategies.

The Xinjiang Uygur Autonomous Region in Northwestern China is one of the country’s largest sheep production bases. Within Xinjiang, the Yili Prefecture is of particular importance. Characterized by a cool, semi-humid continental climate and abundant alpine grasslands, Yili provides ideal environmental conditions for both fine wool sheep breeding and the transmission of trichostrongylid nematodes. Zhaosu and Tekesi are two key pastoral counties within this prefecture, representing the typical transhumance farming systems of the region. Despite the economic importance of the sheep industry here, information regarding the current status of BZ resistance remains scarce and fragmented. Historical data from nearly two decades ago suggested full susceptibility [10], but more recent, limited surveys indicate resistance may be emerging [11]. However, a comprehensive molecular investigation of all three major resistance-associated codons has not yet been conducted specifically in Zhaosu and Tekesi counties of Yili Prefecture. Therefore, this study aims to determine the prevalence of the F167Y, E198A, and F200Y mutations in *H. contortus* populations from these two counties.

Given the heavy reliance on benzimidazoles by local smallholders and the lack of updated resistance data, there is an urgent need to evaluate the efficacy of these drugs. By providing detailed molecular evidence of BZ resistance in this critical region, we aim to offer evidence-based guidance for updating local parasite control programs.

## 2. Materials and Methods

### 2.1. Study Area and Parasite Collection

Adult *Haemonchus contortus* specimens were collected from sheep slaughtered in the counties of Zhaosu and Tekesi, Yili Prefecture, Northern Xinjiang, China. This region is a major hub for fine wool sheep production, characterized by a cool, semi-humid climate that favors nematode transmission. In this area, benzimidazoles (particularly albendazole) are routinely and intensively used by local smallholders for parasite control. However, because the sampled animals originated from various smallholder flocks and were processed at local abattoirs, detailed individual records regarding the timing of the last anthelmintic treatment were unavailable.

Sample collection was conducted between June and July 2025. A total of 40 sheep carcasses were randomly examined at local abattoirs. The geographical origin of each animal was validated using slaughterhouse records and confirmed with the owners. Of the animals examined, 32 abomasa (80%) were found to be naturally infected with nematodes (15 from Zhaosu and 17 from Tekesi). Abomasal contents were washed and screened through sieves. Adult male worms were identified morphologically as *H. contortus* based on standard taxonomic keys [12,13]. From the total worm burden recovered, a subset of 150 adult male worms (75 from each county) was randomly selected, washed repeatedly in physiological saline, and each worm was rinsed at least three times to completely remove intestinal contents, host mucus, and residual feed debris from the body surface, so as to eliminate exogenous DNA contamination from the host and other microorganisms. After cleaning, individual worms were placed into 1.5 mL sterile centrifuge tubes, labeled with sampling location, serial number, and collection date, and then transferred to a −20 °C refrigerator for long-term preservation.

### 2.2. Genomic DNA Extraction

To ensure DNA purity and avoid potential contamination from eggs in female uteri, only adult male worms were used for molecular analysis. Genomic DNA was extracted from each of the 150 individual adult male worms using the DNeasy Blood and Tissue Kit (Qiagen, Hilden, Germany) according to the manufacturer’s protocol, as follows:

(1) Sample Lysis and Pretreatment: Place a single adult worm into a 1.5 mL EP tube, add 200 μL Buffer GA, and vortex thoroughly for resuspension. Add 20 μL Proteinase K solution and mix well. Incubate the sample at 56 °C until the tissues are completely dissolved, then perform a brief centrifugation to remove droplets on the inner wall of the tube cap.
(2) Sample Denaturation and DNA Precipitation: Add 200 μL Buffer GB and mix thoroughly by inverting the tube. Incubate the mixture in a 70 °C water bath for 10 min until the solution becomes clear, then briefly centrifuge to remove residual droplets. Add 200 μL absolute ethanol and shake vigorously for 15 s. After flocculent DNA precipitates form, conduct a short centrifugation.
(3) Column Adsorption and Impurity Washing: Transfer the entire solution and precipitates into the Adsorption Column CB3 placed in a collection tube. Centrifuge at 12,000 rpm (approximately 13,400× *g*) for 30 s and discard the waste liquid. Sequentially add 500 μL Buffer GD and 600 μL Wash Solution PW (confirming that absolute ethanol has been added before use), and centrifuge for 30 s at the same rotating speed. Repeat the washing step with PW once, and discard the waste after each centrifugation.
(4) Air-drying of Residual Reagents: Put the adsorption column back into the collection tube and centrifuge at 12,000 rpm for 2 min to remove residual liquid. Leave the column at room temperature for several minutes to fully evaporate the residual wash solution on the adsorption membrane.
(5) DNA Elution and Collection: Transfer the adsorption column into a new clean centrifuge tube. Pipette 20 μL Elution Buffer TE vertically onto the center of the adsorption membrane, and let it stand at room temperature for 2–5 min. Centrifuge at 12,000 rpm for 2 min. The collected eluate contains purified genomic DNA of adult worms.

After extraction, the quantity and purity of the extracted DNA were assessed using a NanoDrop spectrophotometer (Thermo Fisher Scientific, Waltham, MA, USA). DNA concentration and A260/A280 ratio were strictly controlled between 1.8 and 2.0 for all samples, indicating high purity with no protein or polysaccharide contamination. Samples that failed to meet the purity standard were re-extracted to guarantee the stability and repeatability of downstream PCR experiments. All DNA samples were stored at −20 °C until subsequent PCR amplification.

### 2.3. Molecular Identification

To definitively confirm the morphological identification, the second internal transcribed spacer (ITS-2) region of ribosomal DNA was amplified. PCR was performed on all 150 individual DNA samples using the primers described by Newton et al. [14] (forward: 5′-CAAATGGCATTTGTCTTTTAG-3′; reverse: 5′-TTAGTTTCTTTTCCTCCGCT-3′).

The PCR reaction was carried out in a 25-μL volume containing 2 μL of template DNA, 1 μL of each primer (10 μmol/L), 12.5 μL of 2 × TransStart FastPfu Fly PCR SuperMix (TransGen Biotech, Beijing, China), and 8.5 μL of nuclease-free water. The thermal cycling conditions were as follows: initial denaturation at 94 °C for 5 min, followed by 35 cycles of 94 °C for 30 s, 55 °C for 30 s, and 72 °C for 30 s, with a final extension at 72 °C for 5 min. A no-template negative control was included in every run. Amplicons were separated on 1.0% agarose gels stained with ethidium bromide and visualized under UV light to verify the presence of a single ~265 bp band.

To validate species identity, PCR products from 16 randomly selected individuals (8 from each county) were purified and sequenced bidirectionally by Qingke Biotechnology Co., Ltd. (Wuhan, China). The obtained sequences were aligned and compared with reference sequences in the NCBI GenBank database using BLASTn (https://blast.ncbi.nlm.nih.gov/Blast.cgi, accessed on 23 May 2026).

### 2.4. Amplification and Sequencing of the Isotype-1 Β-Tubulin Gene

A 385 bp fragment of the isotype-1 β-tubulin gene, encompassing the polymorphic sites at codons 167, 198, and 200, was amplified using the primer pair described by Zhang et al. [7]: β-tub-F (5′-GACGCATTCACTTGGAGGAG-3′) and β-tub-R (5′-CATAGGTTGGATTTGTGAGTT-3′).

PCR reactions were prepared in a 25-μL volume using the same reagent concentrations described in Section 2.3. Thermal cycling conditions were also identical to those used for the ITS-2 amplification, except for the annealing temperature (55 °C). A no-template negative control was included in each batch of reactions to monitor contamination. PCR products were visualized on 1.0% agarose gels stained with ethidium bromide. Amplicons of the expected size (385 bp) from all 150 individual worms were purified using a commercial gel extraction kit (Cowin Biotech, Taizhou, China) and sequenced bidirectionally by Qingke Biotechnology Co., Ltd. (Wuhan, China).

### 2.5. Analysis of Benzimidazole Resistance-Associated SNPs

Sequencing chromatograms were manually inspected and analyzed using SnapGene 6.0.2 software. The sequences were screened for specific single nucleotide polymorphisms (SNPs) known to confer benzimidazole resistance: F167Y (TTC→TAC) at codon 167, E198A (GAA→GCA) at codon 198, and F200Y (TTC→TAC) at codon 200.

Genotypes for each individual were scored according to the chromatogram peak patterns:

**Homozygous susceptible (SS)**: Presence of a single peak corresponding to the wild-type nucleotide.

**Homozygous resistant (RR)**: Presence of a single peak corresponding to the mutant nucleotide.

**Heterozygous (RS):** Presence of double peaks at the mutation site, where the secondary peak height exceeded 50% of the primary peak height [15].

The Genotype Frequencies (GF) and Resistant Allele Frequencies (RAF) were calculated for each population using the following formulas:

$$\begin{array}{l} \mathrm{Genotype frequency (GF, \%)}=(\mathrm{number of individuals with a given genotype}/\mathrm{total}\\ \mathrm{number of individuals})\times 100 \end{array}$$

$$\begin{array}{c} \mathrm{Resistant allele frequency (RAF, \%)}=(2\times \mathrm{number of RR individuals}+\mathrm{number of RS}\\ \mathrm{individuals})/(2\times \mathrm{total number of individuals})\times 100 \end{array}$$

## 3. Results

### 3.1. Parasite Collection and Morphological Identification

A total of 1992 adult *H. contortus* worms were recovered from the abomasa of 32 infected sheep (out of the 40 initially examined; 80% prevalence). Specifically, 942 worms were collected from 15 sheep in Zhaosu, and 1050 worms were collected from 17 sheep in Tekesi. The infection intensity varied widely among individuals, ranging from 43 to 425 worms per abomasum.

The high prevalence (80%) and variable worm burden demonstrate that gastrointestinal nematode infection is highly prevalent in local sheep flocks. Severe single-host infection further increases the economic losses caused by anemia, growth retardation, and reduced meat and wool production in sheep. The widespread infection also means that drug-resistant nematode individuals can spread rapidly among flocks through pasture contamination, forming a large resistant population in a short time.

**Figure 1:**
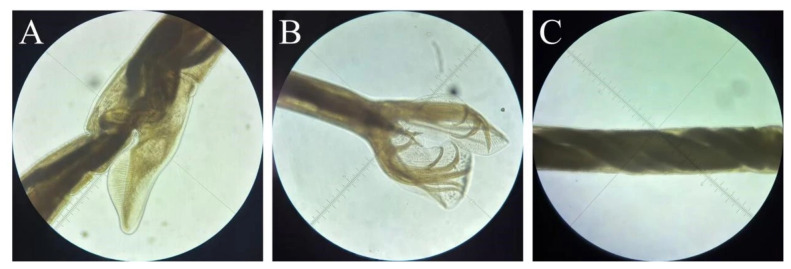
Morphological identification of *Haemonchus contortus* adult worms. (**A**) Characteristic vulval flap of a female worm (linguiform morphotype). (**B**) Y-shaped spicules and copulatory bursa of a male worm. (**C**) Intestine and gonads twisted together Specimens were collected from naturally infected sheep in Yili Prefecture, Xinjiang, China.

Morphological examination confirmed the identity of the specimens as *H. contortus*: female worms exhibited the characteristic linguiform vulval flap (Figure 1 A) and a characteristic “twist” feature (Figure 1C), while males displayed the typical Y-shaped spicules (Figure 1B). From this total collection, a subset of 150 adult male worms (75 from each county) was randomly selected for downstream molecular analysis to ensure representative sampling.

### 3.2. Molecular Confirmation of Species Identity

**Figure 2:**
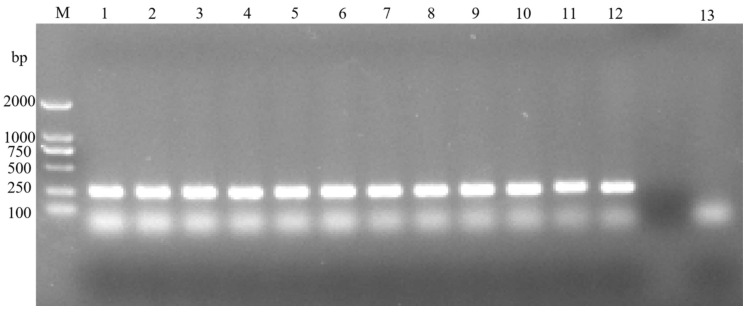
Representative agarose gel electrophoresis of ITS-2 PCR products from adult male Haemonchus contortus. Lanes 1–12: individual worms from Zhaosu (lanes 1–6) and Tekesi (lanes 7–12) counties; Lane 13: no-template negative control; Lane M: 100 bp DNA ladder. All samples produced a single band of the expected size (265 bp).

PCR amplification of the ITS-2 region was successful for all 150 analyzed adult male worms, yielding a single distinct band of approximately 265 bp (Figure 2). To validate the species identity, 16 amplicons (8 from Zhaosu and 8 from Tekesi) were randomly selected for sequencing. BLASTn analysis revealed that all obtained sequences shared 99% nucleotide identity with *H. contortus* reference sequences in GenBank.

### 3.3. Benzimidazole Resistance-Associated SNPs in the Isotype-1 Β-Tubulin Gene

The 385 bp fragment of the isotype-1 β-tubulin gene was successfully amplified and sequenced from all 150 adult male worms.

**Codon 167**: Sequence analysis revealed no polymorphisms at this locus. All 150 individuals from both the Zhaosu and Tekesi populations were homozygous susceptible (TTC/TTC).

**Figure 3:**
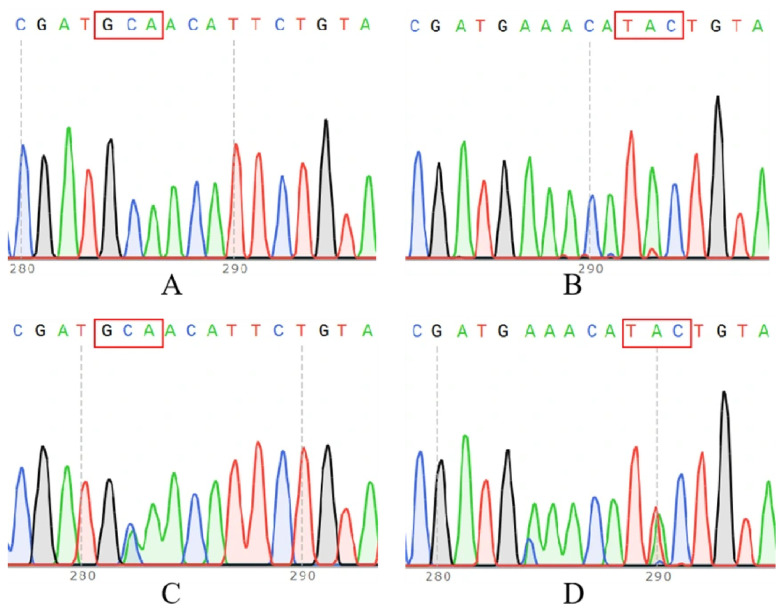
Representative sequencing chromatograms of benzimidazole resistance-associated SNPs in the isotype-1 β-tubulin gene of Haemonchus contortus from sheep in Yili Prefecture, Xinjiang, China. (**A**) Homozygous resistant genotype at codon 198 (GCA, E198A). (**B**) Homozygous resistant genotype at codon 200 (TAC, F200Y). (**C**) Heterozygous genotype at codon 198 (GAA/GCA). (**D**) Heterozygous genotype at codon 200 (TTC/TAC). Polymorphic nucleotides are highlighted in red boxes.

**Codons 198 and 200:** In contrast, resistance-conferring SNPs were detected at both codon 198 (E198A, GAA→GCA) and codon 200 (F200Y, TTC→TAC). As shown in Figure 3, three distinct genotypes were identified: homozygous susceptible (SS), homozygous resistant (RR), and heterozygous (RS). Alignment of the amplified sequences with the wild-type reference gene clearly confirmed the specific nucleotide substitutions at these positions. No additional non-synonymous mutations were observed in the analyzed fragment.

### 3.4. Genotype and Allele Frequencies of Benzimidazole Resistance-Associated SNPs

No mutations were detected at codon 167 (F167Y), with all 150 individuals remaining homozygous susceptible. In contrast, the E198A mutation at codon 198 was exceptionally prevalent and served as the dominant resistance mechanism across both populations. Specifically, in Zhaosu, 44.0% of individuals were homozygous resistant (RR), and 41.3% were heterozygous (RS), leaving only a small fraction of the population susceptible. Similarly, the Tekesi population maintained high levels of E198A, with 32.0% RR and 41.3% RS individuals (Table 1).

Conversely, the distribution of the F200Y mutation at codon 200 exhibited a marked geographical disparity. In Zhaosu, selection for F200Y appeared to be in the early stages, as RR individuals were rare (1.33%) and most mutation carriers were RS (16.0%). However, the F200Y mutation was significantly more prevalent in Tekesi, where it reached a RR rate of 17.3% and a heterozygosity rate of 32.0%, indicating a stronger or more complex selection pressure in this county compared to Zhaosu (Table 1). Heterozygous individuals are considered a transitional state in the evolution of drug resistance; the high proportion of RS genotypes in both populations the continued spread of resistance alleles within the worm population. Compared with Zhaosu, the higher homozygous resistant ratio of F200Y in Tekesi indicates that this locus has undergone longer-term drug selection, and the resistant genotype has gradually become stably inherited in the local population.

### 3.5. Haplotype Analysis at Codons 198 and 200

Haplotype analysis revealed strikingly distinct evolutionary trajectories for the two populations (Table 2). The Zhaosu population was characterized by a single-mechanism resistance profile driven almost exclusively by the E198A mutation, with the HR-198/Hs-200 haplotype (homozygous resistant at 198, susceptible at 200) occurring in 44.0% of individuals. In stark contrast, the Tekesi population exhibited a complex multi-locus resistance pattern indicative of active co-selection. While the E198A-driven haplotype remained common (32.0%), a substantial proportion of the population carried the F200Y-driven haplotype (Hs-198/HR-200, 17.3%) or presented as double-heterozygotes (Het-198/Het-200, 29.3%), suggesting a rapid accumulation of resistance alleles at both loci simultaneously.

### 3.6. Resistant Allele Frequencies

The calculated Resistant Allele Frequencies (RAF) underscore the severity of the resistance status in the region (Table 3). At codon 198 (E198A), the RAF reached extremely high levels in both populations: 64.7% in Zhaosu and 52.7% in Tekesi. In contrast, the RAF at codon 200 (F200Y) revealed a significant divergence in selection pressure between the two counties. While the F200Y allele remained relatively minor in Zhaosu (9.3%), it had risen to substantial levels in Tekesi (33.3%), confirming that the latter population was undergoing a rapid shift towards multi-locus resistance.

## 4. Discussion

This study represents the first comprehensive molecular characterization of benzimidazole (BZ) resistance in *H. contortus* populations from Zhaosu and Tekesi counties in Yili Prefecture, a critical livestock production base in Northwestern China. Our results reveal an alarming resistance landscape characterized by the complete absence of the F167Y mutation, exceptionally high frequencies of the E198A mutation (52.7–64.7%), and moderate-to-high frequencies of F200Y (9.3–33.3%). These values substantially exceed those previously reported from the same region and indicate that BZ resistance has reached a critical threshold, necessitating immediate adjustments to local parasite control strategies.

The coexistence of two major resistance mutations also raises the risk of cross-resistance and multi-drug resistance in local *H. contortus.* Once dual-locus homozygous resistant strains are widely disseminated, benzimidazole drugs will completely lose their deworming effect in the region, and the cost of parasite prevention and control for pastoral farmers will rise sharply. This situation is not unique to Yili; similar trends of rapidly rising BZ resistance have also been reported in other pastoral areas of northern China, suggesting that anthelmintic resistance has become a common challenge restricting the development of the national sheep industry.

A striking finding of this investigation is the predominance of the E198A mutation, which served as the primary driver of resistance in both surveyed populations. This pattern contrasts sharply with the global paradigm of BZ resistance, where the F200Y mutation is typically the dominant mechanism, followed by F167Y [16,17,18,19,20,21,22]. While E198A has historically been considered rare in Europe, the Americas, and Australia, our findings corroborate emerging evidence that this specific mutation has become the predominant BZ-resistance mechanism in *H. contortus* populations across China. The extreme prevalence of E198A observed here (RAF > 50%) ranks among the highest frequencies documented worldwide, suggesting that local selection pressures or specific genetic lineages in Northwestern China heavily favor this mutation.

The temporal evolution of BZ resistance in Yili Prefecture is particularly concerning when viewed against historical baselines. In 2005, surveys conducted in Shihezi and Yili reported a complete absence of resistant homozygotes or heterozygotes at any of the three codons [10]. By 2018, a study in Zhaosu County began to detect early signs of resistance, reporting low frequencies of heterozygotes (11.1%) at codon 198 and rare homozygotes at codon 200 [11]. In stark contrast, the present study documents homozygous-resistant frequencies reaching 44.0% in Zhaosu and 32.0% in Tekesi at codon 198.

This trajectory indicates that *H. contortus* populations in northwestern Xinjiang have transitioned from phenotypically susceptible to highly resistant in less than 20 years. Such a rapid escalation strongly suggests that, despite the lack of specific treatment records for the sampled flocks, the selection pressure exerted by benzimidazoles in this region has been intensive and unrelenting. This acceleration mirrors patterns observed in other high-production agricultural zones and serves as a warning that the efficacy of this drug class is likely compromised, if not completely lost, in the studied areas.

The haplotype analysis further reveals distinct evolutionary trajectories between the two counties. Zhaosu appears to be under a “single-mechanism” selection regime driven almost exclusively by E198A. In contrast, the Tekesi population exhibits a “dual-mechanism” pattern, with significant co-occurrence of E198A and F200Y. Notably, the high prevalence of double heterozygotes (Het-198/Het-200, 29.3%) in Tekesi suggests that resistance alleles at both codons are being maintained simultaneously. This co-selection complicates control efforts, as it increases the genetic reservoir for high-level resistance and potentially accelerates the fixation of double-homozygous resistant genotypes under continued drug pressure.

Future studies should integrate genotypic SNP surveillance with phenotypic efficacy testing, including the egg hatch test (EHT) and the larval development assay (LDA), to directly correlate E198A/F200Y allele frequencies with field benzimidazole treatment outcomes in Yili Prefecture.

## 5. Limitations and Conclusions

We acknowledge that the specific anthelmintic treatment history of the sampled sheep was unavailable due to their origin from various smallholder flocks and abattoirs. However, the exceptionally high frequencies of resistance alleles detected here provide irrefutable molecular evidence of heavy, likely ineffective benzimidazole use in the field. The complete absence of the F167Y mutation suggests that monitoring this specific codon is currently less critical for diagnostics in this region. Given the alarming prevalence of E198A and the emerging dual-resistance pattern in Tekesi, the continued reliance on benzimidazoles in Yili Prefecture is unsustainable. We urgently recommend implementing resistance management strategies, such as the rotation of drug classes (e.g., using macrocyclic lactones or levamisole), targeted selective treatment (TST), and pasture hygiene management to mitigate the further spread of resistant populations [23]. In addition, regular molecular surveillance of drug resistance SNPs should be established as a long-term monitoring mechanism in Yili Prefecture to dynamically track changes in resistance allele frequencies and provide real-time data to support the optimization of regional deworming plans.

## Figures and Tables

**Table 1:** Genotype and resistant allele frequencies of benzimidazole resistance-associated SNPs at codons 198 and 200 in the isotype-1 β-tubulin gene of *Haemonchus contortus* from Zhaosu and Tekesi counties.

Population	Allele	No. of Homozygous Sensitive (%)	No. of Heterozygous (%)	No. of Homozygous Resistant (%)
	167	75 (100)	0	0
Zhaosu	198	11 (14.67)	31 (41.33)	33 (44)
	200	61 (81.33)	12 (16)	2 (2.67)
	167	75 (100)	0	0
Tekesi	198	20 (26.67)	31 (41.33)	24 (32)
	200	38 (50.67)	24 (32)	13 (17.33)

**Table 2:** Haplotype frequencies (%) at codons 198 and 200 of the isotype-1 β-tubulin gene in *Haemonchus contortus* from Zhaosu and Tekesi counties.

Population	Haplotype (Frequencies %)					
	Hs-198 Hs-200	Het-198 Hs-200	Hs-198 Het-200	Het-198 Het-200	Hs-198 HR-200	HR-198 Hs-200
Zhaosu	7 (9.33)	22 (29.33)	3 (4)	9 (12)	1 (1.33)	33 (44)
Tekesi	5 (6.67)	9 (12)	2 (2.67)	22 (29.33)	13 (17.33)	24 (32)

*Hs* homozygous susceptible, *HR* homozygous resistant, *Het* heterozygote.

**Table 3:** Resistant allele frequencies (%) for benzimidazole resistance-associated SNPs in the isotype-1 β-tubulin gene of *Haemonchus contortus* from Zhaosu and Tekesi counties, Yili Prefecture, Xinjiang, China.

Population	Codon 198 (E198A)		Codon 200 (F200Y)	
	Resistant (A)	Susceptible (E)	Resistant (Y)	Susceptible (F)
Zhaosu	64.7	35.3	9.3	90.7
Tekesi	51.3	48.7	33.3	66.7

A: alanine (mutant, resistant); E: glutamic acid (wild-type, susceptible); Y: Tyrosine (mutant, resistant); F: Phenylalanine (wild-type, susceptible).

## Data Availability

The original contributions presented in this study are included in the article/Supplementary Material. Further inquiries can be directed to the corresponding authors.

## Supplementary Materials

The following supporting information can be downloaded at: https://www.mdpi.com/article/10.3390/vetsci13060603/s1.
