# Supplementary figures and images for: Dominance of the E198A Mutation and Emergence of Co-Selection in Benzimidazole-Resistant Haemonchus contortus from Northwestern China
Source: Vet Sci. 2026 Jun 21;13(6):603. doi: 10.3390/vetsci13060603 (PMC13307563; doi:10.3390/vetsci13060603)

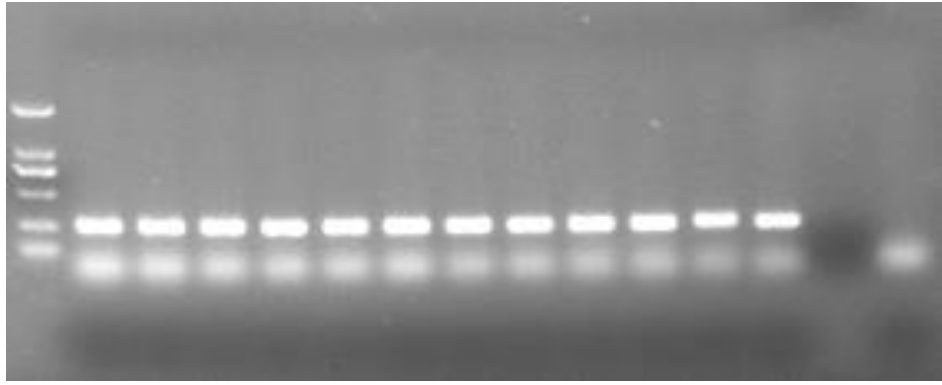

Supplement: Supplementary file 1 [file vetsci-13-00603-s001.zip › vetsci-4366200-supplementary.pdf]
